# Cytolytic replication of echoviruses in colon cancer cell lines

**DOI:** 10.1186/1743-422X-8-473

**Published:** 2011-10-14

**Authors:** Stina Israelsson, Nina Jonsson, Maria Gullberg, A Michael Lindberg

**Affiliations:** 1 School of Natural Sciences, Linnaeus University, SE-391 82 Kalmar, Sweden; 2 Current address: National Veterinary Institute, Division of Virology, Technical University of Denmark, Lindholm, 4771 Kalvehave, Denmark

**Keywords:** enterovirus, echovirus, colon cancer, oncolytic virus, virotherapy

## Abstract

**Background:**

Colorectal cancer is one of the most common cancers in the world, killing nearly 50% of patients afflicted. Though progress is being made within surgery and other complementary treatments, there is still need for new and more effective treatments. Oncolytic virotherapy, meaning that a cancer is cured by viral infection, is a promising field for finding new and improved treatments. We have investigated the oncolytic potential of several low-pathogenic echoviruses with rare clinical occurrence. Echoviruses are members of the enterovirus genus within the family *Picornaviridae*.

**Methods:**

Six colon cancer cell lines (CaCo-2, HT29, LoVo, SW480, SW620 and T84) were infected by the human enterovirus B species echovirus 12, 15, 17, 26 and 29, and cytopathic effects as well as viral replication efficacy were investigated. Infectivity was also tested in spheroids grown from HT29 cells.

**Results:**

Echovirus 12, 17, 26 and 29 replicated efficiently in almost all cell lines and were considered highly cytolytic. The infectivity of these four viruses was further evaluated in artificial tumors (spheroids), where it was found that echovirus 12, 17 and 26 easily infected the spheroids.

**Conclusions:**

We have found that echovirus 12, 17 and 26 have potential as oncolytic agents against colon cancer, by comparing the cytolytic capacity of five low-pathogenic echoviruses in six colon cancer cell lines and in artificial tumors.

## Background

Colorectral cancer is the second and third most commonly diagnosed cancer in the world for women and men respectively. Nearly 50% of those diagnosed with colorectal cancer are killed from it, and in 2008 there were more than 1.2 million new cases estimated of colorectal cancer [1]. Presently the most common treatment of colorectal cancer is surgery, where chemotherapy and radiation may be used as adjuvants [2]. Though progress is made within these therapies, the nearly 50% mortality rate indicates the necessity of new and improved treatments of colorectal cancer.

Viral oncolysis offers a new promising field of cancer treatments. The research area has broadened to include several different mechanisms of antitumoral efficacy; for instance direct tumor cell lysis due to viral replication or viral cytotoxic proteins and transgene expression (reviewed in [3,4]). Many of the oncolytic viruses that have been tested in clinical trials are genetically modified, in particular adenoviruses and herpes simplex virus (reviewed in [5-7]). Several other viruses, including poxviruses, reovirus and Newcastle disease virus have also shown promising antitumor effects in various human malignancies (reviewed in [4,8-10]).

Echoviruses (E) are serotypes belonging in the Human Enterovirus B (HEV-B) species within the enterovirus (EV) genus of *Picornaviridae*. Picornaviruses are small (diameter about 30 nm), naked, icosahedrically shaped viruses with an RNA genome of about 7.2 to 8.5 kb in length with positive polarity [11,12]. Within the EV genus both E1 and coxsackievirus A21 have been shown to be oncolytic in several human cancers, including malignant melanoma and ovarian cancer [13-18]. In addition, it has been reported that bovine EV shows oncolytic characteristics [19], and attenuated, recombinant poliovirus strains have been shown to have great oncolytic potential [20-22]. Recently another picornavirus, Seneca valley virus, has also emerged as an oncolytic virotherapy candidate [23,24].

The HEV-B species contains many viruses with rare clinical occurrence and low pathogenicity, making them potentially suitable for oncolytic virotherapy if they show oncolytic characteristics. The serotypes within the HEV-B species that were chosen to be included in this study of cytolytic capacity were E12, E15, E17, E26, and E29. These viruses were all isolated in the 1950's from healthy persons [25-30] and chosen on the basis of their low pathogenicity [31-34]. In this paper, the cytolytic effects of these five serotypes were investigated in six cell lines derived from cancers of the human colon. Colorectal cancer was chosen due to the fecal-oral route of infectivity of enteroviruses [12] and its position as one of the most common forms of cancer with a mortality rate of nearly 50% [1]. Those serotypes that generated the most efficient cythopathic effect (CPE) and highest amount of viral progeny, in cell lines grown as monolayers, were also tested for infectivity in spheroids (reviewed in [35]). Infection of spheroids enables investigation of the capability to spread within an artificial tumor of three-dimensional shape.

## Materials and methods

### Cell lines and viruses

The colon cancer cell lines HT29, SW480, SW620, LoVo, and T84 were purchased from European Collection of Cell Cultures (ECACC, UK) and CaCo-2 was generously provided by C. Gustafson-Svärd (Linnaeus University, Kalmar, Sweden). A local variant of green monkey kidney (GMK) cells and Chinese hamster ovary (CHO) cells, kindly provided by H.-C. Selinka (University of Tübingen, Tübingen, Germany), were used as positive and negative control for infectivity studies. SW480, SW620, CaCo-2, GMK and CHO were cultured in Dulbeccos modified Eagle's medium (DMEM), HT29 was cultured in McCoy's 5A medium and LoVo and T84 were cultured in Ham's F12 medium. Medias were supplemented with 100 U/ml penicillin, 0.1 mg/ml streptomycin, 2 mM L-glutamine and 10% newborn calf serum (NCS). 5% NCS were used in GMK and CHO media. CaCo-2 media was additionally supplemented with 0.1 mM non-essential amino acids (NEAA). Cell lines were grown in 37°C and 7.5% CO_2 _(HT29, SW480, SW620, GMK and CHO) or 5.0% CO_2 _(CaCo-2, LoVo and T84). E15 *Charleston *was purchased from American Type Culture Collection (ATCC, VR-45), E12 *Travis*, E17 *CHHE-29 *and E26 *Coronel *were generously provided by J.-Å. Liljeqvist, (University of Gothenburg, Gothenburg, Sweden) and E29 *JV-10*, was kindly provided by M. Roivainen (National Institute for Health and Welfare, Helsinki, Finland).

### Viral serotype confirmation

Viral serotypes were confirmed genetically by RNA extraction using QIAamp viral RNA MiniSpinKit (Qiagen) according to manufacturer's manual. RNAs were reverse transcribed (RT) by SuperScript III RT enzyme (Invitrogen) using a primer 5'-ATAAGAATGCGGCCGCT_27_-3' at 50°C for 60 minutes and the enzyme was inactivated at 70°C. cDNAs were amplified by PCR using PuReTaq Ready-To-Go PCR Beads (GE Healthcare) and primers matching the flanking sequences of the VP1 region of each viral strain. Nucleotide sequences were determined using the ABI Prism BigDye terminator cycle sequencing reaction kit (Applied Biosystems) and a 3130 Genetic Analyzer (Applied Biosystems). Sequencher version 4.5 (Gene Codes Corporation) was used for editing of sequences. Obtained viral sequences were determined by strain using basic local alignment search tool (BLAST, National Center for Biotechnology Information, NCBI). Prior to virus infectivity assays all viruses were propagated and titrated in GMK cells using the endpoint titration method of Sperman and Kärber [36].

### Virus infectivity assay

Cells were grown in 6 well plates (HT29, SW480, SW620, LoVo, T84, GMK and CHO) or in T25 bottles (CaCo-2) to a confluence of 50-85%. The cells were washed in phosphate buffered saline (PBS) and serum free medias were added to cells: McCoy's 5A to HT29, DMEM to SW480, SW620, GMK and CHO and OptiMEM to T84, LoVo and CaCo-2. CaCo-2 media was additionally supplemented with 0.1 mM NEAA. Cells were infected with viruses at a multiplicity of infection (MOI) of 1 in double samples and incubated in room temperature for 60 minutes. The virus containing medias were exchanged for new serum free equivalents, and one sample per cell line and virus (E12, E15, E17, E26 or E29) was frozen while the other remaining sample was incubated in 37°C until complete CPE was visible, or for a maximum of seven days. At the time of complete CPE, or after seven days, cells were photographed using microscopy. All infection assays were performed three times. After freezing and thawing three times, all infection samples were titrated in GMK cells.

### Culture of spheroids and spheroid infectivity assay

HT29 spheroids were cultured in 24 well plates by inoculating 1 000 cells per well, coated with 1% agarose in PBS, in 1 ml McCoy's 5A media supplemented with 100 U/ml penicillin, 0.1 mg/ml streptomycin, 2 mM L-glutamine and 10% NCS. Single spheroids were allowed to form for seven days in 37°C, 7.5% CO_2_. 10^5 ^tissue culture infectious dose 50% (TCID_50_) of each virus (E12, E17, E26 or E29) were added to spheroids, and the infections were followed for nine days. Infections were repeated on at least two different occasions and each virus was tested for infectivity in spheroids 14 to 18 times in total.

### Viability confirmation of spheroids

HT29 spheroids, uninfected or infected by E29, were washed separately three times in PBS to remove any single cells or unbound viral particles and dispersed in 0.25% trypsin-EDTA solution. McCoy's media supplemented with 100 U/ml penicillin, 0.1 mg/ml streptomycin, 2 mM L-glutamine and 10% NCS was added. Cells were diluted 1:2 in trypan blue solution, incubated for 10 minutes and examined in a Bürker chamber using microscopy. Trypsinated cells from uninfected spheroids were also allowed to proliferate in 6 well plates for two days.

## Results

### Viral infectivity in colon cancer cell lines

**Figure 1:**
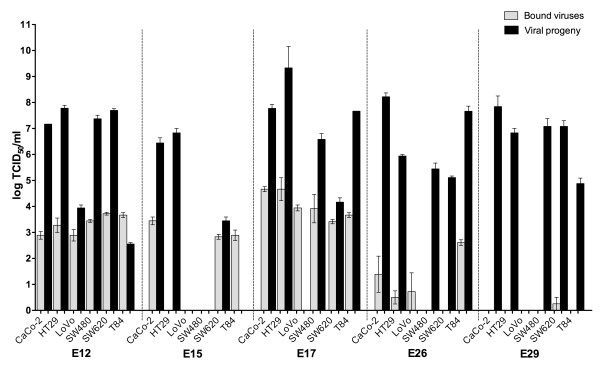
**Viral infections of colon cancer cell lines**. Six colon cancer cell lines: CaCo-2, HT29, LoVo, SW480, SW620 and T84, were infected by either echovirus 12, 15, 17, 26 or 29 in double triplicates. Infections were frozen directly after infection (bound viruses) or incubated until complete CPE was reached, or maximum seven days (viral progeny). All infections were titrated in green monkey kidney cells after having been frozen and thawed three times. Results are presented as mean TCID_50_/ml values ± standard deviation.

To investigate the oncolytic potential of E12, E15, E17, E26 and E29 against colorectal cancer initially, the cytolytic replication in six colon cancer cell lines (Table 1) was analyzed. Viral ability to cause CPE was investigated (Table 2, Additional file 1) as well as increase in viral titers during infection (Figure 1). GMK and CHO cells were used as positive and negative infectious control respectively (data not shown).

**Table 1:** Cell lines derived from human colorectal cancers used in this study

Cell line	Colorectal disease	Tumor stage	Primary tumor/Derived from metastatic site
CaCo-2	adenocarcinoma	-	Primary tumor
HT29	adenocarcinoma	-	Primary tumor
LoVo	adenocarcinoma	Dukes' type C	Left supraclavicular region
SW480	adenocarcinoma	Dukes' type B	Primary tumor
SW620	adenocarcinoma	Dukes' type C	Lymph node
T84	carcinoma	-	Lung

Data are adapted from the American Type Culture Collection (ATCC) catalogue.

-: Tumor stage is not specified.

**Table 2:** Cytopathic effect (CPE) caused by echoviral infection in colon cancer cell lines

Virus	Colon cancer cell line					
	CaCo-2	HT29	LoVo	SW480	SW620	T84
E12	+ (3)	+ (3)	- (7)	+ (2)	+ (3)	+ (3)
E15	+ (7)	+ (3)	- (7)	- (7)	+ (5)	+ (2)
E17	+ (2)	+ (1)	- (7)	+ (6)	+ (7)	+ (1)
E26	+ (5)	+ (2)	- (7)	+/- (7)	+ (7)	+ (2)
E29	+ (3)	+ (2)	- (7)	+ (5)	+ (7)	- (7)

Infections were followed until complete CPE was observed or seven days maximum. Days post infection until complete or partial CPE are indicated within parenthesis. Pictures of infected cells are available in Additional file 1.

+: complete CPE

-: no CPE

+/-: partial CPE

E12 was able to infect all colon cancer cell lines generating an increase in titer of about four logs, 10^3-4 ^to 10^7-8 ^TCID_50_/ml, in HT29, SW480, SW620 and CaCo-2 (Figure 1). The mean titer of E12 attached to LoVo (10^2.89 ^TCID_50_/ml) and that of the viral progeny (10^3.94 ^TCID_50_/ml) indicate that the virus replicated in the cell line, but no CPE was observed after seven days (Table 2, Figure 1). In all other colon cancer cell lines complete CPE was observed within two to three days, although the viral mean titer decreased during the infection in T84.

E15 generated complete CPE in CaCo-2, HT29, SW620 and T84 within two to seven days, but the mean titers of viral progeny, compared to titers of initially attached virus particles, were markedly higher only in the case of HT29 and CaCo-2 (Table 2, Figure 1). In T84, complete CPE was observed after two days, but no viral progeny was measured by titration. No infection was indicated in LoVo or SW480.

In the case of E17, the virus bound to all colon cancer cell lines (Figure 1). In all cell lines except LoVo, complete CPE was observed within one to seven days (Table 2). CPE was also supported by an increase in mean titer between bound virus and viral progeny of about two to four logs in all cases where CPE was observed, except in SW680, where the mean titer increased with less than one log (Figure 1). Although E17 attached to the cellular surface of LoVo (mean initial titer of attached viral particles; 10^3.95 ^TCID_50_/ml), no CPE was observed, nor was any viral progeny detected by endpoint titration after seven days.

E26 was able to cause complete CPE in CaCo-2, HT29, SW620 and T84 within two to seven days. In SW480 only partial CPE was observed after seven days, while no CPE was observed in LoVo after the same time (Table 2). During infection viral mean titers increased about five to seven logs in all infected colon cancer cell lines except LoVo, where no viral progeny was indicated by titration (Figure 1). Mean titers of viral progeny reached about 10^5 ^to 10^8 ^TCID_50_/ml in all cell lines except LoVo.

E29 caused complete CPE in two to seven days in CaCo-2, HT29, SW480 and SW620, generating viral mean titers of about 10^5 ^to 10^8 ^TCID_50_/ml, even though the virus attached to the cellular surfaces of all cell lines in very low amounts (Table 2, Figure 1). E29 also caused an infection in T84, generating a viral progeny mean titer of 10^4.88 ^TCID_50_/ml, but no CPE was observed. LoVo was uninfected by E29.

To summarize these results, E12, E17, E26 and E29 are able to proliferate efficiently and cause cytolytic infection in most of the cell lines included in this study. E15 was excluded at this stage, due to its relatively poor ability to proliferate and cause CPE in the colon cancer cell lines investigated.

### Viral infectivity in HT29 spheroids

**Figure 2:**
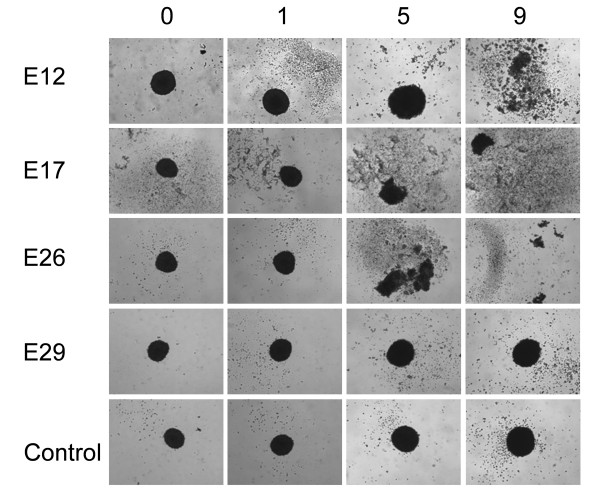
**Viral infections of HT29 spheroids**. Spheroids originating from 1 000 HT29 cells and grown for seven days were infected by 10^5 ^TCID_50 _of echovirus 12, 17, 26, 29 or uninfected (Control). Infections were followed for 9 days and pictures were taken at 0, 1, 5, and 9 days post infection at 4 000 × magnification. The figure shows representative pictures of at least 14 spheroid infections per virus performed on at least two different occasions.

Three-dimensionally *in vitro *growing cells, referred to as spheroids (reviewed in [35]), allow viruses to infect cells in a more tumor-like environment rather than the infection of a bi-dimensional monolayer. Spheroids of the human colon cancer cell line HT29 were infected by E12, E17, E26 and E29. The results show that E12, E17 and E26 destroyed the spheroids within five to nine days (Figure 2). E29 was not able to destroy HT29 spheroids within nine days. Viability of spheroids, both uninfected and infected by E29, was confirmed by individualization of spheroid cells using trypsin treatment, followed by trypan blue staining (data not shown). Viability of uninfected spheroids was additionally confirmed by allowing the individualized cells to proliferate as monolayers (data not shown).

## Discussion

Though improvements are being made, patient prognosis of colorectal cancer is still poor [1]. Presently surgical restriction, chemotherapy and radiation are the most common therapeutic approaches [2]. A promising new field in the battle against cancer is oncolytic virotherapy, which is undergoing extensive investigation. In this study, we investigated the cytolytic capacity of low- or apathogenic echoviruses within the HEV-B species in colon cancer cell lines, grown both as monolayers and as three-dimensional spheroids.

Wild type enteroviruses may be suitable as oncolytic viruses for several reasons. (i) Enteroviruses have been studied epidemiologically for over 50 years and many of these viruses are strictly human pathogens causing low- or even asymptomatic infections [12]. It is therefore well known that there are viruses within this genus with a relatively safe profile from a pathological point of view. This is a favorable characteristic of an oncolytic virus. It should be mentioned though, that low-pathogenic enteroviruses might be associated with more severe symptoms occasionally [37,38]. (ii) Wild type enteroviruses with low pathogenicity can be oncolytic without genetic engineering [16,17]. This eliminates the risks of potential reversion to virulent phenotypes of attenuated oncolytic viruses or loss of malignant tissue specificity as a consequence of genetic engineering. (iii) There are antipicornavirus drugs such as Pleconaril. This drug has been used successfully on a compassionate-release basis [39,40]. (iv) Low-pathogenic enteroviruses may be rare in the human population [16,31]. This decreases the risk of patients having an acquired immunity towards the virus prior to deliberate infection for oncolytic virotherapy purposes. (v) Possibly several different serotypes with similar oncolytic properties within an EV species could be identified. Serotypes could then be altered successively if administrating more than one oncolytic virus to a patient. This would provide a strategy to avoid obstacles due to acquired immunity towards already administered viruses. (vi) The small virion size (about 30 nm in diameter) of picornaviruses [11] could be an oncolytic advantage. Fibrillar collagen has proven to inhibit relatively large herpes simplex virus from reaching malignant cells within a tumor, while nanoparticles spread more easily [41]. (vii) Picornaviruses have a relatively fast replication cycle, ranging from five to ten hours [11]. This could also be an advantage for viral oncolysis, since a faster replicating virus may reduce a tumor in size more effectively than a slower replicating virus [42].

We have investigated the cytolytic capacity of E12, E15, E17, E26 and E29 by infection of six colon cancer cell lines derived from both primary tumors and metastases (Table 1). To be able to address the infectious ability of the viruses in these cell lines as explicitly as possible, no serum was added to the medias in these viral infectivity experiments. The results were recorded both by the ability to cause CPE (Table 2, Additional file 1), and by proliferation efficacy (Figure 1). Highly cytolytic viruses with an efficient propagation were also allowed to infect spheroids (Figure 2), to investigate their ability to spread within and destroy an artificial tumor.

When analyzing the results of the monolayer studies (Table 2, Figure 1) CaCo-2, HT29 and SW620 were cytolytically infected by all viruses, while LoVo was not cytolytically infected by any of the five viruses. However, our results suggest that E12 causes a persistent infection in LoVo. We have not been able to find a likely cause to this infectious disability, and it may be due to both difficulties of viral entry as well as intracellular proliferation factors. A cytolytic infectious cycle within the tumor cells is a favorable characteristic of an oncolytic virus. The release of viral progeny enables spreading to neighboring malignant cells as well as metastatic sites that are distant from primary site of administration. Interestingly, it has been shown that the genetic difference between an EV causing a cytolytic infection and one causing a non-cytolytic infection, in a particular cell line, may be as little as one amino acid change [43].

The viral infections of SW480 and SW620 are interesting, due to the fact that these cell lines originate from the same cancer patient. SW480 was established from a primary adenocarcinoma of the colon, while SW620 was established from a lymph node metastasis one year later (Table 1). The cell lines seem to respond differently to infection of the same virus. E26 does not cause cytolysis in SW480 to the same extent that it does in SW620 (Table 2). The mean viral progeny of E17 in SW620 is about 10^4 ^TCID_50_/ml, while the mean viral progeny in SW480 is more than 10^6 ^TCID_50_/ml (Figure 1). E12 and E29 cause CPE faster in SW480 than in SW620, and even though E15 is not able to infect SW480, the virus causes a cytolytic infection in SW620 (Table 2, Figure 1). This indicates that the same viral serotype may have a different impact when infecting cancer cells derived from the same original cancer. Hence, there may be a "sliding therapeutic window" in the progression of a cancer, where a specific oncolytic virus has the most effective anti-cancer effect. In this perspective, several oncolytic viral serotypes with similar oncolytic properties may be very beneficial, not only from an immunological point of view.

All viruses cause a cytolytic infection in T84 except E29, which seems to cause a non-cytolytic infection (Table 2, Figure 1). Strangely, the viral progeny titers of E12 and E15 are lower than the initial titers in T84 (Figure 1). This could to some extent be explained by viral progeny neutralization caused by neutralizing substances released by lysed cells, or less viable progeny production of E12 and E15 in T84. More viral particles adsorbing to the T84 cellular surface than the number of particles entering and infecting the cells could also contribute to this effect. In the case of E12, it is known that decay-accelerating factor (DAF or CD55) is a cellular receptor [44,45]. This receptor may not be enough for cellular entry, hence infection, for DAF-binding picornaviruses [46]. It is possible that unknown entry-receptors of E12 and E15 are not expressed on the surface of T84 or expressed in very low amounts. In addition, viral attachment-induced apoptosis could contribute to the CPE observed in E12 and E15 infected T84 cells. It has been shown that high MOIs of UV-inactivated reovirus virions can induce apoptosis in the absence of viral replication by interacting with cell surface receptors [47]. Due to the inefficient cytolytic replication of E15, this virus was excluded from further studies.

In addition to confirmation of successful lytic infections and measurement of viral progeny following infection, an interesting pattern emerges when comparing binding titers of the different viruses in Figure 1. Several serotypes generate similar binding titers independent of cell line infected. A MOI of 1 was used in the experiment and the time required for complete CPE is not connected to initial binding titers (Table 2). The titers of attached E12 and E17 are relatively high, while the amount of bound E29 (and in some cases E26 and E15) are not detected in all cell lines. This could be the result of different entry strategies. If viruses with very low or undetected binding titers use a highly specific attachment and entry process, infection could be successful even at undetected initial amount of bound virions using the TCID_50 _method. This method does not tell how many infectious particles are present in the original sample, but what dilution of virus will generate CPE in 50% of the cells inoculated [36]. A high titer of bound viruses indicates that relatively many virions interact with the cell surface initially. This could be an effect of a more virion dense attachment and entry strategy, possibly by the use of more than one receptor.

E12, E17, E26 and E29 were further investigated in spheroids, cells grown in three-dimensional clusters, hence more resembling tumors than cells grown as monolayers. The results (Figure 2) show that E12, E17 and E26 were able to dissolve spheroids of HT29 cells grown for seven days, starting with 1 000 cells. E29 was not able to efficiently kill HT29 cells when grown as spheroids. These cells were only readily infected and lysed by E29 when grown as monolayers (Table 2, Figure 1). The viability of E29 infected and uninfected spheroids were confirmed after seven days by trypsination and trypan blue staining. Trypan blue staining showed that E29 had some cytolytic effect on the spheroids, but not enough to dissolve them. Almost all cells from uninfected spheriods were viable after seven days (data not shown). The viability of uninfected spheroids was also confirmed by letting individualized spheroid cells proliferate as monolayers again (data not shown). These results show that even though a virus may cause cytolysis in a certain cell population when grown as a monolayer, the same virus may not be as infectious when the cells are grown in three-dimensional clusters.

A major concern regarding all oncolytic viruses is the existence of neutralizing antibodies from prior natural or deliberate exposure. Employing low- or apathogenic viruses for oncolytic purposes raises the question whether these viruses are frequently circulating in the human population without causing any symptoms although inducing immunity. Because they are not frequently associated with disease there are very little data published regarding circulation of these viruses in the population. The seroprevalence of the oncolytic candidates we present here needs to be addressed in coming studies. However, seroprevalence of E1 and coxsackievirus A21, two other low-pathogenic oncolytic enteroviruses, seems to be less than 10% in preliminary studies [16,48]. Besides investigation of the potential risk of preexisting neutralizing antibodies, this study will need to be complemented with infection studies in primary cell cultures and animal models. This would further elucidate the oncolytic capacity of these viruses. Knowledge of infection preferences, for example receptor usage, of these viruses would also be a great advantage when investigating the ability to utilize these viruses for oncolytic purposes. E12 and E29 have been shown to utilize DAF (CD55) as cellular receptor [44,45,49]. DAF is a vital part of directing the immune system away from self-tissue [50], and has been shown to be upregulated in colon cancer [51,52]. In addition to *in vivo *studies of the oncolytic capacity, the receptor usage of these viruses needs to be further elucidated while bearing in mind that enteroviruses may utilize more than one receptor [46,53]. Our study pinpoints E12, E17 and E26 as new potential candidates for oncolytic virotherapy. It also puts focus on a group of enteroviral serotypes that have not been extensively studied before, due to the fact that they are not causing serious diseases or financial losses to the human population. However, seen in the light of oncolytic virotherapy, these viruses may be of great interest as new treatments in the battle against cancer.

## Conclusions

In conclusion, the echoviruses 12, 17 and 26 show potential as oncolytic agents against colon cancer. This was found by investigating the cytolytic capacity and replicative efficiency of E12, E15, E17, E26 and E29 in six colon cancer cell lines as well as in artificial HT29 tumors *in vitro*.

## Competing interests

The authors declare that they have no competing interests.

## Authors' contributions

SI planned the experimental setup, prepared viral stocks, carried out viral infectivity assays and cultured and infected spheroids. SI wrote the manuscript. NJ participated in preparation of virus stocks and viral infectivity assays. MG participated in titrating viral stocks. AML was involved in the study design and revision of the manuscript. All authors have read and approved the final manuscript.

## Supplementary Material

Additional file 1 **Pictures of infected colon cancer cell lines**. CaCo-2, HT29, LoVo, SW480, SW620 and T84 infected with echovirus 12, 15, 17, 26, 29 (at a multiplicity of infection of 1) and uninfected (Control). Pictures were taken at the time of complete cytopathic effect or seven days post infection at 4 000 or 10 000 × magnification. Click here for file

## References

[B1] Jemal A Bray F Center MM Ferlay J Ward E Forman D Global cancer statistics CA Cancer J Clin 2011 61 69 90 10.3322/caac.20107 21296855

[B2] Blumberg D Ramanathan RK Treatment of colon and rectal cancer J Clin Gastroenterol 2002 34 15 26 10.1097/00004836-200201000-00005 11743241

[B3] Mullen JT Tanabe KK Viral oncolysis Oncologist 2002 7 106 119 10.1634/theoncologist.7-2-106 11961194

[B4] Liu TC Galanis E Kirn D Clinical trial results with oncolytic virotherapy: a century of promise, a decade of progress Nat Clin Pract Oncol 2007 4 101 117 10.1038/ncponc0736 17259931

[B5] Ko D Hawkins L Yu DC Development of transcriptionally regulated oncolytic adenoviruses Oncogene 2005 24 7763 7774 10.1038/sj.onc.1209048 16299536

[B6] Mathis JM Stoff-Khalili MA Curiel DT Oncolytic adenoviruses - selective retargeting to tumor cells Oncogene 2005 24 7775 7791 10.1038/sj.onc.1209044 16299537

[B7] Shen Y Nemunaitis J Herpes simplex virus 1 (HSV-1) for cancer treatment Cancer Gene Ther 2006 13 975 992 10.1038/sj.cgt.7700946 16604059

[B8] Kirn DH Thorne SH Targeted and armed oncolytic poxviruses: a novel multi-mechanistic therapeutic class for cancer Nat Rev Cancer 2009 9 64 71 10.1038/nrc2545 19104515

[B9] Kim M Chung YH Johnston RN Reovirus and tumor oncolysis J Microbiol 2007 45 187 192 17618222

[B10] Ravindra PV Tiwari AK Sharma B Chauhan RS Newcastle disease virus as an oncolytic agent Indian J Med Res 2009 130 507 513 20090097

[B11] Racaniello V Knipe M, Howley PM Picornaviridae: The viruses and their replication Fields Virology 2007 1 5 Philadelphia: Lippincott Williams and Wilkins, a Wolters Kluwer business 795 838

[B12] Pallansch M Roos R Knipe M, Howley PM Enteroviruses: polioviruses, coxsackieviruses, echoviruses and newer enteroviruses Fields Virology 2007 1 5 Philadelphia: Lippincott Williams and Wilkins, a Wolters Kluwer business 839 893

[B13] Au GG Lincz LF Enno A Shafren DR Oncolytic Coxsackievirus A21 as a novel therapy for multiple myeloma Br J Haematol 2007 137 133 141 10.1111/j.1365-2141.2007.06550.x 17391493

[B14] Berry LJ Au GG Barry RD Shafren DR Potent oncolytic activity of human enteroviruses against human prostate cancer Prostate 2008 68 577 587 10.1002/pros.20741 18288643

[B15] Haley ES Au GG Carlton BR Barry RD Shafren DR Regional administration of oncolytic Echovirus 1 as a novel therapy for the peritoneal dissemination of gastric cancer J Mol Med 2009 87 385 399 10.1007/s00109-008-0433-0 19139835

[B16] Shafren DR Au GG Nguyen T Newcombe NG Haley ES Beagley L Johansson ES Hersey P Barry RD Systemic therapy of malignant human melanoma tumors by a common cold-producing enterovirus, coxsackievirus a21 Clin Cancer Res 2004 10 53 60 10.1158/1078-0432.CCR-0690-3 14734451

[B17] Shafren DR Sylvester D Johansson ES Campbell IG Barry RD Oncolysis of human ovarian cancers by echovirus type 1 Int J Cancer 2005 115 320 328 10.1002/ijc.20866 15688406

[B18] Skelding KA Barry RD Shafren DR Systemic targeting of metastatic human breast tumor xenografts by Coxsackievirus A21 Breast Cancer Res Treat 2009 113 21 30 10.1007/s10549-008-9899-2 18256929

[B19] Smyth M Symonds A Brazinova S Martin J Bovine enterovirus as an oncolytic virus: foetal calf serum facilitates its infection of human cells Int J Mol Med 2002 10 49 53 12060850 10.3892/ijmm.10.1.49

[B20] Gromeier M Lachmann S Rosenfeld MR Gutin PH Wimmer E Intergeneric poliovirus recombinants for the treatment of malignant glioma Proc Natl Acad Sci USA 2000 97 6803 6808 10.1073/pnas.97.12.6803 10841575 PMC18745

[B21] Ochiai H Moore SA Archer GE Okamura T Chewning TA Marks JR Sampson JH Gromeier M Treatment of intracerebral neoplasia and neoplastic meningitis with regional delivery of oncolytic recombinant poliovirus Clin Cancer Res 2004 10 4831 4838 10.1158/1078-0432.CCR-03-0694 15269159

[B22] Toyoda H Yin J Mueller S Wimmer E Cello J Oncolytic treatment and cure of neuroblastoma by a novel attenuated poliovirus in a novel poliovirus-susceptible animal model Cancer Res 2007 67 2857 2864 10.1158/0008-5472.CAN-06-3713 17363609

[B23] Reddy PS Burroughs KD Hales LM Ganesh S Jones BH Idamakanti N Hay C Li SS Skele KL Vasko AJ Yang J Watkins DN Rudin CM Hallenbeck PL Seneca Valley virus, a systemically deliverable oncolytic picornavirus, and the treatment of neuroendocrine cancers J Natl Cancer Inst 2007 99 1623 1633 10.1093/jnci/djm198 17971529 PMC5261858

[B24] Wadhwa L Hurwitz MY Chevez-Barrios P Hurwitz RL Treatment of invasive retinoblastoma in a murine model using an oncolytic picornavirus Cancer Res 2007 67 10653 10656 10.1158/0008-5472.CAN-07-2352 18006805

[B25] Hammon WM Ludwig EH Pavia RA McCloskey LW Sather GE Problems raised by certain ECHO viruses in the attempted laboratory detection of poliomyelitis virus infection Ann N Y Acad Sci 1957 67 304 310 10.1111/j.1749-6632.1957.tb46054.x 13411968

[B26] Ormsbee RA Melnick JL Biologic and serologic characteristics of ECHO viruses from West Virginia J Immunol 1957 79 384 392 13491847

[B27] Ramos-Alvarez M Sabin AB Intestinal viral flora of healthy children demonstrable by monkey kidney tissue culture Am J Public Health Nations Health 1956 46 295 299 10.2105/AJPH.46.3.295 13292578 PMC1623578

[B28] Wigand R Sabin AB Antigenic purity and plaque properties of the prototype strains of ECHO virus types 7 to 11, and 17 and 18 Arch Gesamte Virusforsch 1962 11 708 717 10.1007/BF01243309 14040255

[B29] Hammon WM Yohn DS Pavia RA Isolation and characterization of prototype viruses ECHO-26, ECHO-27, Coxsackie B-6 Proc Soc Exp Biol Med 1960 103 164 168 14399342 10.3181/00379727-103-25446

[B30] Rosen L Kern J Bell JA Observations On A Group Of Viruses (Jv-5, Jv-6 And Jv-10) Comprising A Newly Recognized Enterovirus Serotype Am J Hyg 1964 79 7 15 14117623 10.1093/oxfordjournals.aje.a120365

[B31] Khetsuriani N Lamonte-Fowlkes A Oberst S Pallansch MA Enterovirus surveillance--United States, 1970-2005 MMWR Surveill Summ 2006 55 1 20 16971890

[B32] Trallero G Avellon A Otero A De Miguel T Perez C Rabella N Rubio G Echevarria JE Cabrerizo M Enteroviruses in Spain over the decade 1998-2007: virological and epidemiological studies J Clin Virol 2010 47 170 176 10.1016/j.jcv.2009.11.013 20007023

[B33] Trallero G Casas I Tenorio A Echevarria JE Castellanos A Lozano A Brena PP Enteroviruses in Spain: virological and epidemiological studies over 10 years (1988-97) Epidemiol Infect 2000 124 497 506 10.1017/S0950268899003726 10982074 PMC2810936

[B34] Tseng FC Huang HC Chi CY Lin TL Liu CC Jian JW Hsu LC Wu HS Yang JY Chang YW Wang HC Hsu YW Su IJ Wang JR Epidemiological survey of enterovirus infections occurring in Taiwan between 2000 and 2005: analysis of sentinel physician surveillance data J Med Virol 2007 79 1850 1860 10.1002/jmv.21006 17935170

[B35] Santini MT Rainaldi G Three-dimensional spheroid model in tumor biology Pathobiology 1999 67 148 157 10.1159/000028065 10394136

[B36] Hierholzer JC Killington RA Mahy BWJ, Kangro HO Virus isolation and quantitation Virology Methods Manual 1996 Glasgow: Academic press limited 25 46

[B37] Bahri O Rezig D Nejma-Oueslati BB Yahia AB Sassi JB Hogga N Sadraoui A Triki H Enteroviruses in Tunisia: virological surveillance over 12 years (1992-2003) J Med Microbiol 2005 54 63 69 10.1099/jmm.0.45695-0 15591257

[B38] Dhole TN Ayyagari A Chowdhary R Shakya AK Shrivastav N Datta T Prakash V Non-polio enteroviruses in acute flaccid paralysis children of India: vital assessment before polio eradication J Paediatr Child Health 2009 45 409 413 10.1111/j.1440-1754.2009.01529.x 19712176

[B39] De Palma AM Vliegen I De Clercq E Neyts J Selective inhibitors of picornavirus replication Med Res Rev 2008 28 823 884 10.1002/med.20125 18381747

[B40] Rotbart HA Webster AD Treatment of potentially life-threatening enterovirus infections with pleconaril Clin Infect Dis 2001 32 228 235 10.1086/318452 11170912

[B41] McKee TD Grandi P Mok W Alexandrakis G Insin N Zimmer JP Bawendi MG Boucher Y Breakefield XO Jain RK Degradation of fibrillar collagen in a human melanoma xenograft improves the efficacy of an oncolytic herpes simplex virus vector Cancer Res 2006 66 2509 2513 10.1158/0008-5472.CAN-05-2242 16510565

[B42] Wodarz D Viruses as antitumor weapons: defining conditions for tumor remission Cancer Res 2001 61 3501 3507 11309314

[B43] Gullberg M Tolf C Jonsson N Polacek C Precechtelova J Badurova M Sojka M Mohlin C Israelsson S Johansson K Bopegamage S Hafenstein S Lindberg AM A single coxsackievirus B2 capsid residue controls cytolysis and apoptosis in rhabdomyosarcoma cells J Virol 2010 84 5868 5879 10.1128/JVI.02383-09 20375176 PMC2876633

[B44] Bergelson JM Chan M Solomon KR St John NF Lin H Finberg RW Decay-accelerating factor (CD55), a glycosylphosphatidylinositol-anchored complement regulatory protein, is a receptor for several echoviruses Proc Natl Acad Sci USA 1994 91 6245 6248 10.1073/pnas.91.13.6245 7517044 PMC44175

[B45] Powell RM Schmitt V Ward T Goodfellow I Evans DJ Almond JW Characterization of echoviruses that bind decay accelerating factor (CD55): evidence that some haemagglutinating strains use more than one cellular receptor J Gen Virol 1998 79 Pt 7 1707 1713 9680134 10.1099/0022-1317-79-7-1707

[B46] Shafren DR Dorahy DJ Ingham RA Burns GF Barry RD Coxsackievirus A21 binds to decay-accelerating factor but requires intercellular adhesion molecule 1 for cell entry J Virol 1997 71 4736 4743 9151867 10.1128/jvi.71.6.4736-4743.1997 PMC191695

[B47] Tyler KL Squier MKT Rodgers SE Schneider BE Oberhaus SM Grdina TA Cohen JJ Dermondy Differences in the capacity of reovirus strains to induce apoptosis aredetermined by the viral attachment protein sigma 1 J Virol 1995 69 6972 6979 7474116 10.1128/jvi.69.11.6972-6979.1995 PMC189616

[B48] Karttunen A Pöyry T Vaarala O Ilonen J Hovi T Roivainen M Hyypiä T Variation in enterovirus receptor genes J Med Virol 2003 70 99 108 10.1002/jmv.10352 12629650

[B49] Ward T Pipkin PA Clarkson NA Stone DM Minor PD Almond JW Decay-accelerating factor CD55 is identified as the receptor for echovirus 7 using CELICS, a rapid immuno-focal cloning method Embo J 1994 13 5070 5074 7525274 10.1002/j.1460-2075.1994.tb06836.x PMC395453

[B50] Lublin DM Atkinson JP Decay-accelerating factor: biochemistry, molecular biology, and function Annu Rev Immunol 1989 7 35 58 10.1146/annurev.iy.07.040189.000343 2469439

[B51] Koretz K Bruderlein S Henne C Moller P Decay-accelerating factor (DAF, CD55) in normal colorectal mucosa, adenomas and carcinomas Br J Cancer 1992 66 810 814 10.1038/bjc.1992.365 1384641 PMC1977964

[B52] Nakagawa M Mizuno M Kawada M Uesu T Nasu J Takeuchi K Okada H Endo Y Fujita T Tsuji T Polymorphic expression of decay-accelerating factor in human colorectal cancer J Gastroenterol Hepatol 2001 16 184 189 10.1046/j.1440-1746.2001.02418.x 11207899

[B53] Israelsson S Gullberg M Jonsson N Roivainen M Edman K Lindberg AM Studies of Echovirus 5 interactions with the cell surface: heparan sulfate mediates attachment to the host cell Virus Res 2010 151 170 176 10.1016/j.virusres.2010.05.001 20466025

