# Supplementary figures and images for: Cytolytic replication of echoviruses in colon cancer cell lines
Source: Virol J. 2011 Oct 14;8:473. doi: 10.1186/1743-422X-8-473 (PMC3213228; doi:10.1186/1743-422X-8-473)

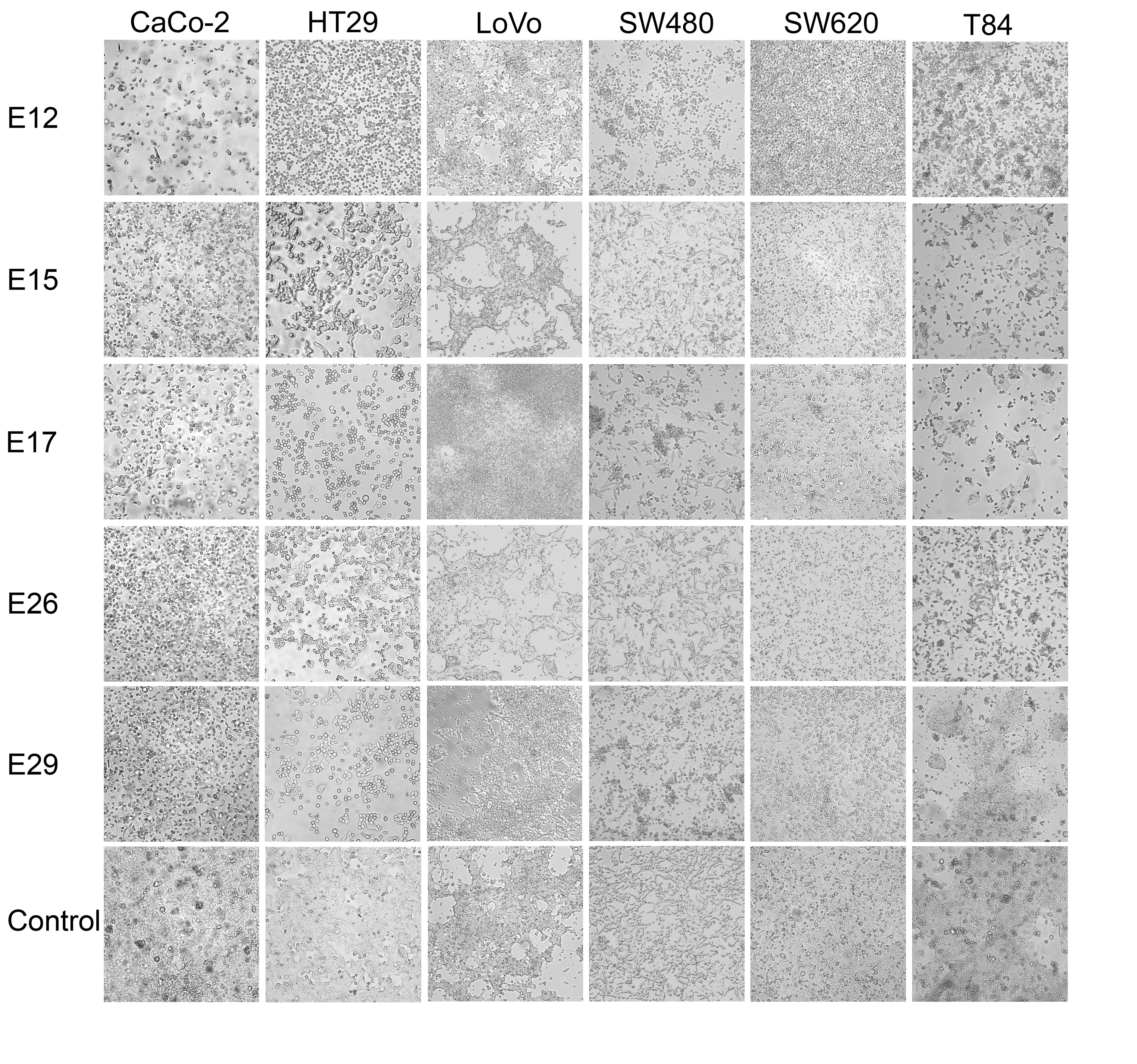

Supplement: Additional file 1 — Pictures of infected colon cancer cell lines. CaCo-2, HT29, LoVo, SW480, SW620 and T84 infected with echovirus 12, 15, 17, 26, 29 (at a multiplicity of infection of 1) and uninfected (Control). Pictures were taken at the time of complete cytopathic effect or seven days post infection at 4 000 or 10 000 × magnification. [file 1743-422X-8-473-S1.JPEG]
